# Intratumoral and Peritumoral Radiomics of Contrast-Enhanced CT for Prediction of Disease-Free Survival and Chemotherapy Response in Stage II/III Gastric Cancer

**DOI:** 10.3389/fonc.2020.552270

**Published:** 2020-12-04

**Authors:** Junmeng Li, Chao Zhang, Jia Wei, Peiming Zheng, Hui Zhang, Yi Xie, Junwei Bai, Zhonglin Zhu, Kangneng Zhou, Xiaokun Liang, Yaoqin Xie, Tao Qin

**Affiliations:** ^1^ Department of Gastrointestinal Surgery, Zhengzhou University People’s Hospital, Henan Provincial People’s Hospital, Henan University People’s Hospital, Zhengzhou, China; ^2^ Department of Ophthalmology, Henan Key Laboratory for Ophthalmology, Henan Provincial People’s Hospital, Henan Provincial Ophthalmology Hospital, Zhengzhou, China; ^3^ Department of Clinical Laboratory, Henan Provincial People’s Hospital, Zhengzhou University People’s Hospital, Henan University People’s Hospital, Zhengzhou, China; ^4^ School of Computer and Communication Engineering, University of Science and Technology Beijing, Beijing, China; ^5^ Shenzhen Institutes of Advanced Technology, Chinese Academy of Sciences, Shenzhen, China; ^6^ Shenzhen Colleges of Advanced Technology, University of Chinese Academy of Sciences, Shenzhen, China; ^7^ Department of Hepatobiliary Pancreatic Surgery, Zhengzhou University People’s Hospital, Henan Provincial People’s Hospital, Henan University People’s Hospital, Zhengzhou, China

**Keywords:** gastric cancer, radiomics signature, computed tomography, prognosis, support vector machine

## Abstract

**Background:**

We evaluated the ability of radiomics based on intratumoral and peritumoral regions on preoperative gastric cancer (GC) contrast-enhanced CT imaging to predict disease-free survival (DFS) and chemotherapy response in stage II/III GC.

**Methods:**

This study enrolled of 739 consecutive stage II/III GC patients. Within the intratumoral and peritumoral regions of CT images, 584 total radiomic features were computed at the portal venous-phase. A radiomics signature (RS) was generated by using support vector machine (SVM) based methods. Univariate and multivariate Cox proportional hazards models and Kaplan-Meier analysis were used to determine the association of the RS and clinicopathological variables with DFS. A radiomics nomogram combining the radiomics signature and clinicopathological findings was constructed for individualized DFS estimation.

**Results:**

The radiomics signature consisted of 26 features and was significantly associated with DFS in both the training and validation sets (both *P*<0.0001). Multivariate analysis showed that the RS was an independent predictor of DFS. The signature had a higher predictive accuracy than TNM stage and single radiomics features and clinicopathological factors. Further analysis showed that stage II/III patients with high scores were more likely to benefit from adjuvant chemotherapy.

**Conclusion:**

The newly developed radiomics signature was a powerful predictor of DFS in GC, and it may predict which patients with stage II and III GC benefit from chemotherapy.

## Background

Gastric cancer (GC) is the fifth most commonly diagnosed malignancy and ranks third in cancer-related deaths worldwide (1). Most patients in China are diagnosed at an advanced stage, and surgical resection is the main curative method for GC (2, 3). For patients with advanced GC, prognosis remains dismal even after radical resection, with approximately 20% experiencing relapse within 1 year of the initial surgery (4, 5). Thus, the high rate of tumor recurrence in patients with advanced GC highlights the importance of considering adjuvant treatments (5, 6). However, the survival rates for many stage II and III patients were still low though initial high response rates (4, 5). Thus, it is highly necessary to develop a precise classification of GC that could be applied to better predict survivals and chemotherapy responses for GC patients.

Computed tomography (CT) imaging could give more comprehensive information of tumor heterogeneity than focal tissue samples, and the emerging field of radiomics has great potential for facilitating better clinical decision-making (7, 8). In recent years, radiomics has been increasingly utilized to extract and analyze quantitative imaging features, such as textural heterogeneity, intensity distributions, shape descriptors, and spatial relationships (8). Radiomic methods have been applied to predict the diagnosis, prognosis, therapeutic response, and underlying genomic patterns in several types of tumors (7, 9–12). Several explorative studies have investigated the potential of radiomics in predicting outcomes in GC (10, 13, 14). However, whether radiomic features have value in the prediction of disease-free survival (DFS) and chemotherapy response in patients with stage II and III GC is still unclear and controversial.

State-of-the-art classification algorithms such as support vector machines (SVMs) could be applied to select a small subgroup of discriminating features and patients attributes to construct reliable disease classifiers (15, 16). SVM was introduced by Vapnik (17) for data classification and function approximation. In recent years, SVM has been introduced to solve various biomedical problems (18–20). Hence, the aim of this study was to develop an SVM-based RS to estimate DFS and to assess its predictive value to chemotherapy benefits in patients with stage II/III GC.

## Methods

### Study Design and Patient Cohorts

In this study, we collected data from a total of 739 patients with GC (**Figure S1**). For the training set, data were obtained from 286 patients treated with radical gastrectomy between January 2007 and December 2010 in Henan Provincial People’s Hospital at Zhengzhou University (Zhengzhou, China). Patients were included on the basis of the following criteria: histologically confirmed GC; no other concurrent malignant neoplasms; standard unenhanced and contrast-enhanced abdominal CT performed <7 days before surgical resection; harvested lymph nodes >15; and perioperative, pathological and follow-up data was available; and on other concurrent malignant tumor. *These patients were excluded* if the primary tumor could not be identified on CT, or if patients had received anticancer treatment preoperitive. We also included 453 patients, with the same selection criteria as above, who were treated between January 2011 and December 2012 in Henan Provincical People’s Hospital at Zhengzhou University (Zhengzhou, China) as the validation cohort. The patients were followed up with abdominal CT scans every 6–12 months for the first 2 years after surgery and then annually thereafter. According to the 8th edition of the American Joint Committee on Cancer (AJCC) Cancer Staging Manual of the AJCC/International Union Against Cancer, the TNM staging was restaged (21). The informed consent requirement was signed. The studies involving human participants were reviewed and approved by ethics committee of Henan Provincial People’s Hospital.

### CT Image Acquisition and Processing

All patients underwent contrast-enhanced abdominal CT scans prior to surgery. Portal venous-phase CT images were extracted from the picture archiving and communication system (PACS) (Carestream, Canada). Details of the CT acquisition parameters and image retrieval procedure are described in the **Supplementary Materials**. The primary tumor was manually delineated on the CT images using ITK-SNAP software (www.itksnap.org) by two radiologists in consensus (with 5 and 6 years of clinical experience in abdominal CT interpretation). Any discrepancies were resolved by a third radiologist (11 years of experience in abdominal CT interpretation). Both radiologists were blinded to the clinical and histopathological data but knew the patients had GC. To capture information in the invasive margin, a peripheral ring surrounding the primary tumor was created with automated dilation of the tumor boundaries by 2 mm on the outside and shrinkage of the tumor boundaries by 1 mm on the inside, resulting in a ring with a thickness of 3 mm (22). Large vessels, air cavities, and adjacent organs were excluded.

### Image Feature Extraction

We calculated a total of 584 features from each region of interest (ROI) of each patient’s CT image to characterize peritumoral and intratumor heterogeneity and complexity. For each ROI, i.e., peritumoral and intratumoral areas, we extracted a total of 292 quantitative features. The image features included 14 first-order intensity features, 8 shape features, and 270 second- and higher-order textural features, which are summarized in the **Supplementary Materials**. In this study, we extracted four types of texture features, namely, gray-level co-occurrence matrix (GLCM), gray-level run length matrix (GLRLM), gray-level size zone matrix (GLSZM), and neighborhood gray-tone difference matrix (NGTDM) features, as well as wavelet decomposition features. A Laplacian Gaussian spatial bandpass filter (^∇2^G) was used to derive image features at different spatial scales by turning the filter parameter between 1.0 and 2.5 (1.0, 1.5, 2.0, 2.5). All features were calculated in MATLAB R2012a (The MathWorks Inc.) using an open-source radiomic analysis package (https://github.com/mvallieres/radiomics/). The detailed mathematical definitions of all features are presented in the **Supplementary Materials**.

### Development of the SVM-Based RS

SVM is a binary classifier trained on a group of labeled patterns called training samples (23). The aim of training an SVM is to get a hyperplane that separates the samples into two sides so that all the points with the same label could be on the same side of the hyperplane (15, 17, 19, 24, 25). In this study, we used a two-class classification problem (i.e., whether a patient recurred within 5 years). The SVM-recursive feature elimination (RFE) method was adopted for feature selection and ranking using the training dataset (15). To examine the possibility of identifying different risk subgroups of patients based on these radiomic features using SVM, we performed a set of experiments in the training cohort of 286 patients; then, the SVM-based radiomic classifier was further validated in 453 patients in the validation cohort. In the training cohort, patients on the side of the hyperplane who had more relapses were classified as having low RS score. The SVM data processing methods were conducted as previously described (15, 18, 19, 25). The programs were coded using R software (version 3.4.2). The performance of SVM was evaluated by the sensitivity, specificity, and area under the receiver operating characteristic (ROC) curve (AUC).

### Integrated Nomogram Construction

We constructed an integrated nomogram for the individualized assessment of DFS by combining the imaging signature and clinicopathological factors. Harrell’s concordance index (C-index) was applied to evaluate the accuracy of the model for prognostic prediction (26). We also assessed the overall performance with prediction error curves (PECs) over time and the integrated Brier score (IBS) (27). To quantify the relative improvement in prediction accuracy, the net reclassification improvement (NRI) was calculated. Decision curve analysis (DCA) was performed to quantify the net benefit at various threshold probabilities (28).

### Statistical Analysis

We compared two groups using t-test for continuous variables and χ2 test or Fisher’s exact test for categorical variables, as appropriate. Survival curves for different variable values were generated using the Kaplan-Meier method and were compared using the log-rank test. Variables that reached significance with *P* < 0.05 were entered into the multivariable analyses using the Cox regression model. Interactions between the classifier and chemotherapy were evaluated by means of the Cox model as well. Calibration plots were generated to explore the performance characteristics of the nomogram. DCA was used to evaluate the clinical usefulness of the nomograms. The nomograms and calibration plots were generated with the rms package of R software. All statistical analyses were performed using R software (version 3.4.2) and SPSS software (version 22.0). All statistical tests were two-sided, and *P* < 0.05 was considered to be statistically significant.

## Results

### Patient Characteristics

The detailed clinicopathological characteristics of the patients in the training cohort (n=286) and validation cohort (n=453) are listed in **Table 1**. Of the 739 patients, 515 (68.6%) were men, and the median (interquartile range [IQR]) age of all patients was 58.0 (50.0–65.0) years. The patients in the training cohort and validation cohort were balanced for DFS, with a median (IQR) DFS of 43.0 (29.0–65.0) months for the training cohort and 40.0 (26.0–67.0) months for the validation cohort (log-rank *P* = 0.246), and for the baseline clinicopathologic factors (**Table 1**). **Table S1** shows the association between the RS and clinicopathological variables in the training cohort and validation cohort.

**Table 1:** Clinical characteristics of the patients in the training and validation cohorts.

Variables	Training cohort (N=286)		Validation cohort (N=453)		*P*-value
	No.	%	No.	%	
**Sex**					0.650
Female	87	37.50%	145	62.50%	
Male	199	39.25%	308	60.75%	
**Age(years), median(IQR)**	57(50–65)		58(50–65)		
**Age(years)**					0.805
<60	163	39.09%	254	60.91%	
≧60	123	38.20%	199	61.80%	
**Tumor size(cm)**					0.051
<4	107	43.67%	138	56.33%	
≧4	179	36.23%	315	63.77%	
**Tumor location**					0.436
Cardia	119	40.61%	174	59.39%	
Body	61	41.78%	85	58.22%	
Antrum	95	35.85%	170	64.15%	
Whole	11	31.43%	24	68.57%	
**Differentiation status**					0.195
Well+Moderate	54	43.90%	69	56.10%	
Poor and undifferentiated	232	37.66%	384	62.34%	
**Lauren type**					0.019
Intestinal type	111	44.58%	138	55.42%	
Diffuse or mixed type	175	35.71%	315	64.29%	
**CEA**					0.221
Normal	215	37.52%	358	62.48%	
Elevated	71	42.77%	95	57.23%	
**CA199**					0.001
Normal	240	41.88%	333	58.12%	
Elevated	46	27.71%	120	72.29%	
**Depth of invasion**					0.035
T1	7	43.75%	9	56.25%	
T2	30	52.63%	27	47.37%	
T3	84	42.00%	116	58.00%	
T4a	141	34.14%	272	65.86%	
T4b	24	45.28%	29	54.72%	
**Lymph node metastasis**					0.928
N0	59	36.65%	102	63.35%	
N1	58	40.00%	87	60.00%	
N2	58	36.94%	99	63.06%	
N3a	76	40.00%	114	60.00%	
N3b	35	40.70%	51	59.30%	
**Stage**					0.741
II	102	37.92%	167	62.08%	
III	184	39.15%	286	60.85%	
**Chemotherapy**					0.456
No	152	40.00%	228	60.00%	
Yes	134	37.33%	225	62.67%	

### RS-SVM and Survival

**Figure 1:**
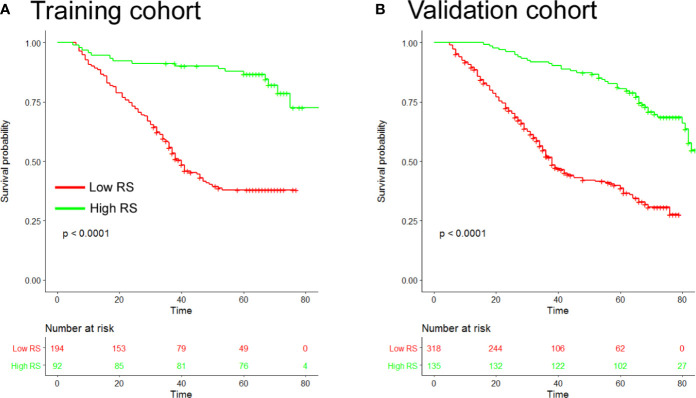
Kaplan-Meier analyses of disease-free survival (DFS) according to the radiomic signature in patients with gastric cancer **(A)**. Training cohort (n=286) **(B)**, Validation cohort (n=453). RS, radiomic signature.

Based on the SVM analysis of the training data, the RS-SVM signature integrated 26 predictors, including 18 intratumoral features and 8 margin features. The features were shown in the **Supplementary Materials**. In the training cohort, there was a significant difference in DFS between patients with low and high-RS scores (hazard ratio (HR) 0.190 (95% confidence interval (CI) 0.112–0.324); P<0.0001; **Table S2**). The 5-year DFS rates for the low-RS score patients was 25.3%, and that for the high RS score patients was 82.6% (**Figure 1A**). To confirm the association between the RS and prognosis, we tested it in the validation cohort and found similar results for DFS [HR 0.252 (95% CI 0.177–0.360); P<0.0001; **Table S2**]. The 5-year DFS rate for the low RS patients was 19.5%, and that for the high RS score patients was 75.6% (**Figure 1B**). In univariate analysis, low RS score patients were associated with significantly poorer DFS (**Table S2**). Multivariate Cox regression analysis after adjustment for clinicopathological risk factors and TNM stage showed that the RS remained an independent predictor of DFS in the training cohort [HR 0.190 (95% CI 0.112–0.323); P<0.0001], as well as in the validation cohort [HR 0.240 (0.168–0.343); P<0.0001; **Table 2**].

**Table 2:** Multivariable cox regression analysis of radiomics signature (RS), TNM stage, and survival in the training and validation cohorts.

Variable	Disease-free Survival	
	HR (95% CI)	*P-*value
**Training cohort (N=286)**		
RS (high vs. low)	0.190(0.112–0.323)	<0.0001
Stage (III vs. II)	2.073(1.389–3.096)	<0.0001
		
**Validation cohort (N=453)**		
RS (high vs. low)	0.240(0.168–0.343)	<0.0001
Stage (III vs. II)	3.249(2.366–4.461)	<0.0001
CA199 (high vs. low)	1.421(1.078–1.872)	0.013

DFS, Disease-free survival.

To further explore whether RS can stratify patients in different stage, we evaluated the prognostic value of RS in patients with stage II and stage III GC (**Figure S1**). Stage II or stage III GC patients with high RS scores had a significantly longer DFS than patients with low RS scores. Moreover, when stratified by other clinicopathological variables such as location, size, differentiation, and histology, the RS was still a statistically significant prognostic classifier in the subgroups, suggesting its independent prognostic value (**Figure S2**).

**Figure 2:**
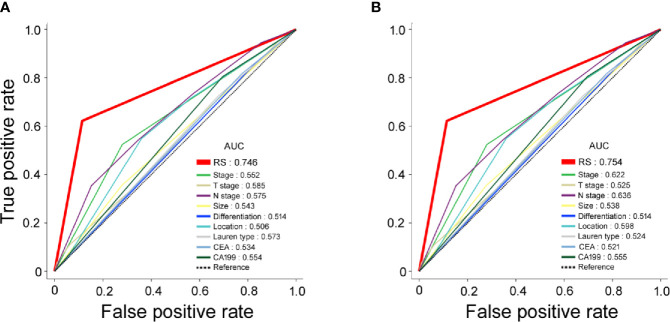
Receiver operating characteristic (ROC) curves for radiomic signature (RS), clinical stage, clinicopathological characteristics as predictors of 5-year disease-free survival (DFS) in the training and validation cohorts. **(A)** Training cohort; **(B)** validation cohort.

The ROC curves for the RS and traditional clinicopathological prognostic factors, including age, sex, CEA, CA19-9, differentiation, tumor size, Lauren type, and TNM stage, illustrated the point with the maximum AUC for each factor. In the subset of evaluated patient cases in each cohort, the AUCs of the RS for 5-year DFS (training cohort: 0.746; validation cohort: 0.754; **Figure 2**) were significantly higher than the AUCs for all other clinicopathological factors considered (next largest was AUC for TNM stage, training cohort: 0.552; validation cohort: 0.622). In addition, the AUC values of the RS were higher than any single radiomic feature included in the RS in the training and validation cohorts (**Figure S3**).

### Nomogram Integrating RS-SVM Signature and Clinicopathologic Factors

**Figure 3:**
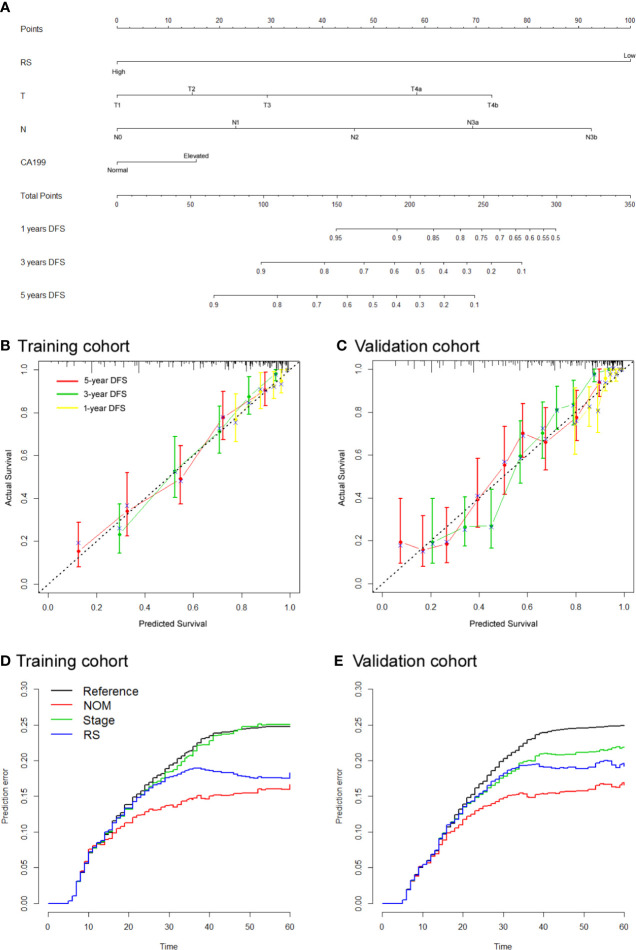
Use of the constructed radiomics nomogram to estimate disease-free survival (DFS) for gastric cancer (GC), along with the calibration and prediction error curves **(A)**. Radiomics nomogram to estimate DFS. Calibration curves for the radiomics nomogram of DFS in the training cohort **(B)** and validation cohort **(C)** show the calibration of each model in terms of the agreement between the estimated and the observed 1-, 3-, and 5-year survival outcomes. The nomogram-estimated DFS is plotted on the x-axis, and the observed DFS is plotted on the y-axis. The diagonal dotted line is a perfect estimation by an ideal model, in which the estimated outcome perfectly corresponds to the actual DFS. The solid line is the performance of the nomogram: a closer alignment with the diagonal dotted line represents a better estimation **(D, E)**. Prediction error curves for each model. Lower prediction errors indicate higher model accuracy.

To assess patient prognosis, we generated a nomogram (**Figure 3A**) for DFS in the training cohort by integrating RS and 3 clinicopathological risk factors, including depth of invasion, lymph node metastasis, and CA-199 level, which were significantly associated with DFS. The calibration curves of the nomogram at 1, 3, and 5 years showed good agreement between the actual and the estimated DFS in the training and validation cohorts (**Figures 3B, C**). The C-index of the nomogram was significantly higher than that of TNM stage (0.768 (0.740–0.795) vs. 0.639 (0.612–0.665), *P*<0.001 in the validation cohort). We computed the NRI for the integrated nomogram vs. stage, which showed significantly improved prediction performance for the nomogram, with an NRI of 0.520 (95% CI 0.417–0.652; *P* < 0.001) in the training cohort, and 0.416 (0.301–0.505; *P* < 0.001) in the validation cohort. The PEC of the nomogram, stage and RS are shown in **Figures 3D, E**. The IBS for the nomogram and stage were 0.124 and 0.160, respectively, in the training cohort and 0.115 and 0.149 in the validation cohort. DCA graphically demonstrated that the nomogram provided larger net benefit across the range of reasonable threshold probabilities than the TNM staging system (**Figure S4**).

### RS-SVM and Benefit From Adjuvant Chemotherapy

**Figure 4:**
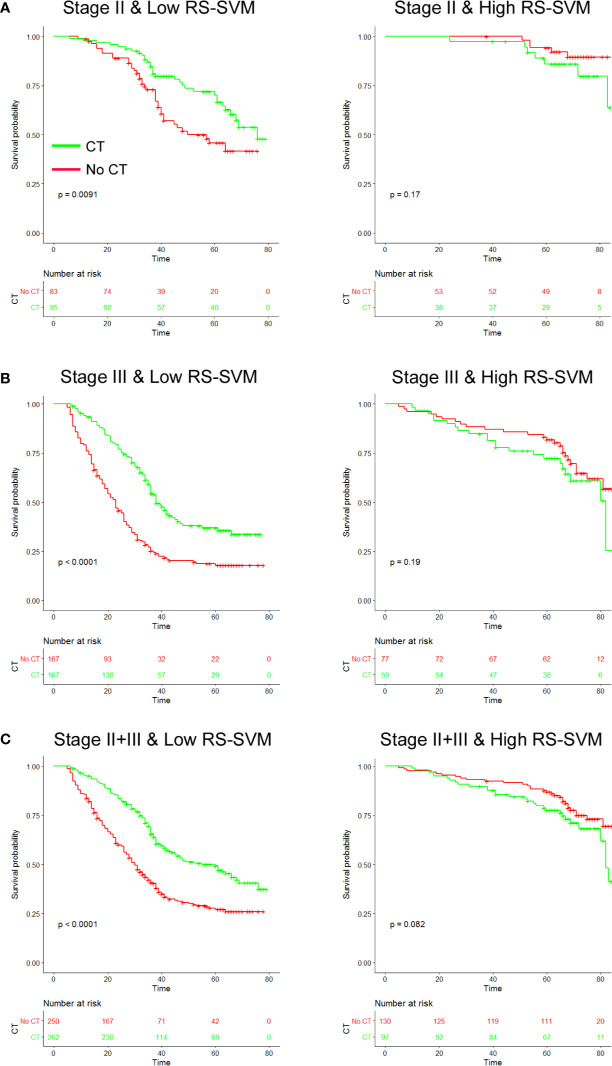
Chemotherapy benefits in gastric cancer compared using disease-free survival (DFS). Kaplan-Meier survival curves for patients with gastric cancer in different radiomics score subgroups, which were stratified by the receipt of chemotherapy **(A)**. Training cohort (n=286) **(B)**, validation cohort (n=453), and **(C)** combined cohort (n=739). CT, chemotherapy; RS, radiomics score.

To investigate whether high or low RS score patients might benefit from adjuvant chemotherapy, we evaluated the association between RS score and DFS among stage II and III patients who either received or did not receive adjuvant chemotherapy. The characteristics of patients who received chemotherapy were similar to those of patients who did not receive adjuvant chemotherapy (**Table S3**). The corresponding Kaplan–Meier survival curves for patients with stage II or stage III disease, which comprehensively compared low with high RS by adjuvant chemotherapy, are shown in **Figure 4**. In High RS score group, there was no significant difference between patients who received chemotherapy and who did not receive chemotherapy for DFS (**Figure 4**). For patients who did or did not receive chemotherapy, RS was associated with DFS in the training and validation cohorts (**Figure S5**). High RS scores seemingly had a greater association with the DFS of patients who received chemotherapy than patients who did not receive chemotherapy (**Figure S5**). Hence, we did a subgroup analysis according to RS score.

We found that chemotherapy was associated with improved prognosis in the low RS score group for both stage II and III GC, [stage II: HR 0.537 (0.333–0.865), P=0.011; stage III: HR 0.469 (0.360–0.612), P<0.001; **Table 3**]. However, for patients in the high RS score group, adjuvant chemotherapy did not affect DFS in either stage II or III GC [stage II: HR 2.194 (0.695–6.920), P=0.18; stage III: HR 1.145 (0.823–2.568), P=0.198]. We performed a formal interaction test between the RS and adjuvant chemotherapy, which confirmed a significant interaction regarding the impact on DFS in stage II GC (*P*=0.030 for interaction, **Table 3**) and stage III GC (*P*=0.001 for interaction).

**Table 3:** Treatment interaction with radiomics signature (RS) for DFS in patients with gastric cancer.

RS	CT	No CT	Disease-free Survival		
			CT vs No CT,	*P*	*P* value for
			HR (95% CI)		interaction
**Stage II (n = 269)**					
RS high	38	53	0.537(0.333–0.865)	0.011	0.030
RS-SVM low	95	83	2.194(0.695–6.920)	0.18	
**Stage III (n = 470)**					
RS high	59	77	0.469(0.360–0.612)	<0.001	0.001
RS-SVM low	167	167	1.145(0.823–2.568)	0.198	
**Stage II+III (n = 739)**					
RS high	97	130	0.411(0.247–0.686)	0.001	<0.0001
RS low	262	250	1.562(0.941–2.594)	0.085	

CT, chemotherapy; DFS, disease-free survival.

## Discussion

Accurate assessment of prognosis is vital for risk stratification and the formation of appropriate treat strategies. GC is a clinically heterogeneous disease, with large variations in outcomes even among GC patients with the same stage (29, 30). Therefore, we wanted to improve the prediction of DFS by building a new RS-SVM model to classify patients into different subgroups with large differences in DFS. Multivariable Cox regression analysis demonstrated that the RS was an independent predictor of DFS, even after adjustment for TNM stage and clinicopathological variables. Moreover, the RS reinforced the prognostic ability of TNM stage, thereby adding prognostic value to TNM staging. By combining clinicopathological and imaging predictors, we showed that the integrated nomogram had a much improved prognostic accuracy compared with TNM staging. These results demonstrate that the imaging signature provided useful complementary information about patient prognosis beyond currently known clinicopathological predictors. Considering the wide availability and routine use of CT scans in clinical practice, this approach will have positive implications for the management of patients with GC.

Extensive studies have suggested the importance of radiomics in cancers and its correlation with prognosis (8, 12, 14, 31, 32). In this study, we attempted to apply a novel combined intratumoral and peritumoral radiomics approach for predicting DFS. Therefore, we created a peripheral ring with automated dilatation of the tumor boundaries by 2 mm on the outside and shrinkage of the tumor boundaries by 1 mm on the inside, resulting in a ring with a thickness of 3 mm. Peritumoral radiomics might provide unique and valuable features, that may reflect peritumoral immune cell infiltration (31, 33, 34). Chen et al. found that the combined intratumoral and peritumoral radiomics model had a better predictive performance of the immunoscore than the intratumoral radiomics model (33). Ferté suggested that combined intratumoral and peritumoral radiomics was a promising way to predict CD8 cell infiltration and to infer clinical outcomes for cancer patients who had been treated with anti-PD-1 and PD-L1 (32). Jiang et al. built an ImmunoScore of gastric cancer (IS_GC_) based on 5 immune features in the invasive margin and center of the tumor, and the IS_GC_ could effectively predict survival and identify patients who might benefit from chemotherapy (3, 35). Khorrami et al. showed that the shape and texture features extracted from the intratumoral and peritumoral regions of lung tumors on CT images could identify patients with pathological response to neoadjuvant chemoradiation (36). In addiction, peritumoral radiomic features were also associated with pathologic immune response (31).

At present, the standard treatment for advanced GC includes adjuvant chemotherapy after surgery to prevent disease recurrence and improve survival; however, many studies have reported that a subgroup of patients could not benefit from adjuvant chemotherapy (5, 12, 18, 37, 38). Moreover, the criteria for the selection of candidates who are more likely to benefit from adjuvant chemotherapy remain controversial. Thus, the accurate identification of subgroups of patients will improve the prognostic system and lead to more personalized therapy. Recently, several studies reported that radiomics signatures based on CT/MRI/PET images were associated with chemotherapy response in several types of cancers (14, 39–41). Jiang et al. developed a 19-feature RS from the intratumoral region of CT images using the lasso-Cox model that, could identify patients with different prognoses and may select chemosensitive patients (13). In addition, Braman et al (39). evaluated radiomic features extracted from peritumoral and intratumoral tissues in the context of neoadjuvant chemotherapy for breast cancer and found that intratumoral and peritumoral radiomics features could strongly predict pathologic complete response (PCR) independent of the choice of classifier. In this study, our RS combined intratumoral and peritumoral radiomics features, and could identify patients more likely chemotherapy. We found that adjuvant chemotherapy provided a more survival benefit to patients classified as having low RS score, whereas those classified as having a high RS score did not obtain benefits from adjuvant chemotherapy; further use of the RS may allow for better identification of patients who are most likely to benefit from adjuvant therapy. Thus, we think that patients with low RS scores may be treated with new combinations of more tolerable medication as an adjunct to potentiate the efficacy of systemic approaches. Therefore, our CT image-based RS for patients with stage II and III GC is both a prognostic and predictive tool, in these patients with low RS scores have a clear benefit from adjuvant chemotherapy. It is worth noting that in western countries, patients with locally advanced gastric cancer typically receive neoadjuvant or perioperative chemotherapy instead of adjuvant chemotherapy after surgery (42, 43). Because the proposed radiomic signature may reflect the biological characteristics, we expect it to be applicable in neoadjuvant or perioperative settings. The mechanism of the association between the CT image-based RS and chemotherapy response has not been shown thoroughly, and further investigation into this relationship may provide additional targets and strategies for treatment.

Our study has several limitations. First, it was a retrospective analysis that suffers from inherent biases. Second, the decision of whether to treat patients with adjuvant chemotherapy after surgery was made by the clinicians. This may limit our predictive analysis using randomized treatment despite the use of a propensity score matching strategy. Third, all CT images were obtained from single-vendor CT scanners (GE); thus, our results need further validation with other CT vendors to check for generalizability. In the current study, the primary tumor was manually delineated on the CT scans by radiologists, which is a challenging and time-consuming task. Development of advanced machine learning methods for semi or fully automated tumor segmentation may facilitate its wide implementation in the future. For enhanced practical acceptability, several aspects including auto-segmentation, feature implementation, and streamlined calculation of RS will be essential. Finally, the model was developed and validated by data from East Asian patients, and its generalizability in Western populations remains to be determined. Ideally, a prospective, randomized clinical trial including both Asian and non-Asian populations will be needed to validate our results findings.

In conclusion, we developed and validated an SVM-based RS that can effectively predict DFS, which provided additional prognostic value to the traditional staging system. In addition, the RS may be a useful tool to predict which patients could benefit from adjuvant chemotherapy. These results warrant further validation in future randomized trials to test the clinical utility of the imaging signature in combination with clinicopathologic criteria to guide individual treatment.

## Data Availability Statement

The original contributions presented in the study are included in the article/**Supplementary Material**, further inquiries can be directed to the corresponding author/s.

## Ethics Statement

The informed consent requirement was signed. The studies involving human participants were reviewed and approved by ethics committee of Henan Provincical People’s Hospital.

## Author Contributions

JL and TQ conceptualized and designed the study.: HZ, ZZ, YX, and JB collected and assembled the data CZ, JW, KZ, XL, and YX analyzed and interpreted the data. All authors wrote the manuscript. All authors contributed to the article and approved the submitted version.

## Funding

This study was supported by the project of the Medical Service Capacity Improvement of Henan Province (2019), the Key Research And Development of Henan Province (Scientific and Technological Research, 2021) and the Youth Project of the National Natural Science Foundation of China (No.81802094).

## Conflict of Interest

The authors declare that the research was conducted in the absence of any commercial or financial relationships that could be construed as a potential conflict of interest.

## Abbreviations

AUC, area under the ROC curve; DFS, disease-free survival; GC, gastric cancer; PACS, picture archiving and communication system; ROC, receiver operating characteristic; ROI, region of interest; RS, radiomics signature; RS-SVM, radiomic signature of support vector machine; SVM, support vector machine.
