# Supplementary material for: Intratumoral and Peritumoral Radiomics of Contrast-Enhanced CT for Prediction of Disease-Free Survival and Chemotherapy Response in Stage II/III Gastric Cancer
Source: Front Oncol. 2020 Dec 4;10:552270. doi: 10.3389/fonc.2020.552270 (PMC7794018; doi:10.3389/fonc.2020.552270)
Supplement: Supplementary file 1 [file DataSheet_1.pdf]

Supplementary Materials

I. Supplementary methods

II. Supplementary reference

III. Supplementary figures

IV. Supplementary tables

V. Definition of image features

1. CT image acquisition and retrieving procedure

All patients underwent contrast-enhanced abdominal CT using the multidetector row CT (MDCT) systems (64-section LightSpeed VCT, GE Medical Systems, Milwaukee, Wis). The acquisition parameters are as follows: 120 kV; 150-190 mAs; 0.5- or 0.4-second rotation time; detector collimation: 64×0.625 mm; field of view, 350×350 mm; matrix, 512×512. After routine non-enhanced CT, arterial and portal venous-phase contrast-enhanced CT were performed after delays of 28 s and 60 s following intravenous administration of 90 - 100 ml of iodinated contrast material (Ultravist 370, Bayer Schering Pharma, Berlin, Germany) at a rate of 3.0 or 3.5 ml/s with a pump injector (Ulrich CT Plus 150, Ulrich Medical, Ulm, Germany). Contrast-enhanced CT was reconstructed with a reconstruction thickness of 2.5 mm.

Portal venous phase CT images (thickness: 2.5 mm) were retrieved from the picture archiving and communication system (PACS) (Carestream, Canada) for image feature extraction because of well differentiation of the tumor tissue from the adjacent normal bowel wall.

2. Image Processing

We analyzed the portal venous-phase CT images because of well differentiation between the tumor tissue and adjacent normal bowel wall. The relatively coarse and heterogeneous resolution in z-axis compared with in-plane resolution would not allow a meaningful and reliable 3D analysis of the image. Therefore, we focused on the most representative 2D slice, i.e., largest tumor section in the axial plane. Two radiologists (with 5 and 6 years of clinical experience in abdominal CT interpretation,

respectively) manually delineated the primary tumor on the CT images by using the ITK-SNAP (http://www.itksnap.org). Both radiologists were blinded to the clinical and histopathological data but were aware that the patients had gastric cancer. All tumor contours were delineated by the two radiologists in consensus.

3. The SVM based Radiomic Signature

SVM, a method for building a classifier, aims to create a decision boundary between two classes that enables prediction of labels from one or more feature vectors.[1] This decision boundary, known as the hyperplane, is orientated in such a way that it is as far as possible from the closest data points from each of the classes. These closest points are called support vectors. In this study, we addressed the DFS prediction problem of GC as a classification problem where the input was a vector that we call a “pattern” of n components which were called “features”. The features consisted of 584 radiomics feature in total. Each pattern corresponds to a patient. We limited ourselves to a two-class classification problem (i.e., whether a patient’s DFS was longer than 5 years or not). The recursive feature elimination method was applied for features selection and ranking in the training dataset.[2] The pruning method was applied to exclude useless features. On the basis of the SVM analysis in the training data of 286 patients, the RS-SVM signature integrated 26 predictors, including 8 margin features and 18 intratumoral features. The 8 margin features were CTmax, CTmedian, GLSZM_LZLGE, NGTDM_Strength, GLSZM_GLN_1.5, GLSZM_LZE_2.0, GLSZM_GLN_2.0, GLRLM_RP_2.5. The 18 margin features were GLCM_ClusterShade, GLCM_InverseVariance, GLCM_MaximumProbability,

GLSZM_ZSN, GLSZM_LGZE, NGTDM_Contrast, GLCM_DifferenceVariance_1.0, GLCM_IMC2_1.0, GLRLM_LRE_1.0, GLCM_DifferenceEntropy_1.5, GLCM_Correlation_2.0, GLRLM_RP_2.0, GLRLM_LGRE_2.0, GLRLM_LRLGE_2.0, GLSZM_ZSV_2.0, GLCM_IMC2_2.5, NGTDM_Contrast_2.5, NGTDM_Complexity_2.5.

4. R Software Packages Used for Statistical Analysis

Survival analysis was performed with the “survival” package. Multivariate Cox regression, nomograms, and calibration plots were generated with the “rms” package. Comparisons between C-indexes were performed with the “Hmisc” package. The “survIDINRI” package was used for the calculation of net reclassification improvement. Decision curve analysis was performed with the function of “dca.R”. Reported statistical significance levels were all two-sided. The statistical significance level was set at 0.05.

Reference

1. Noble WS. What is a support vector machine? Nat Biotechnol 2006; 24: 1565-1567.
2. Wang HY, Sun BY, Zhu ZH et al. Eight-signature classifier for prediction of nasopharyngeal [corrected] carcinoma survival. J Clin Oncol 2011; 29: 4516-4525.

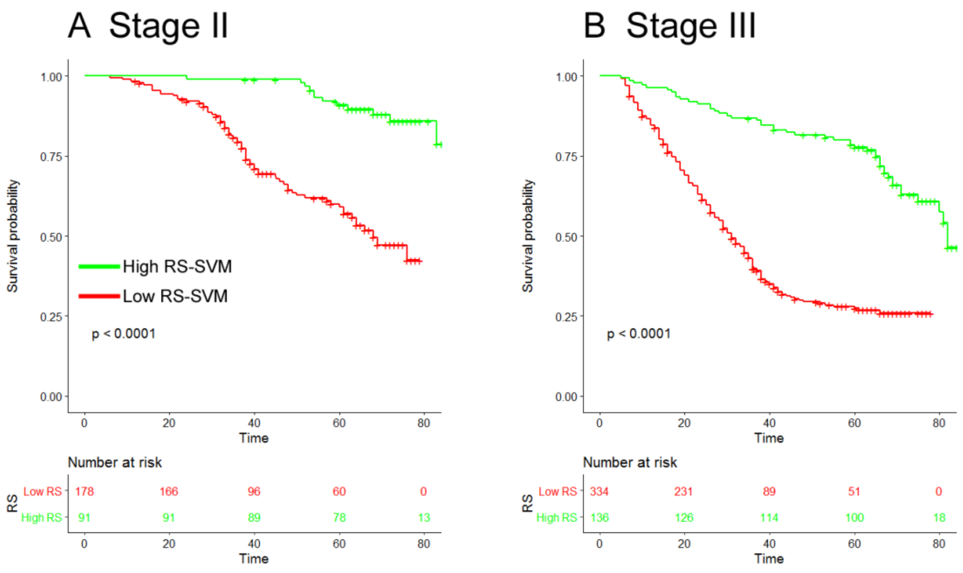

Figure S1. Kaplan-Meier survival analyses of disease-free survival according to the dichotomized RS in stage II and III patients. A, Stage II (*n*=269); (B) Stage III (*n*=470).

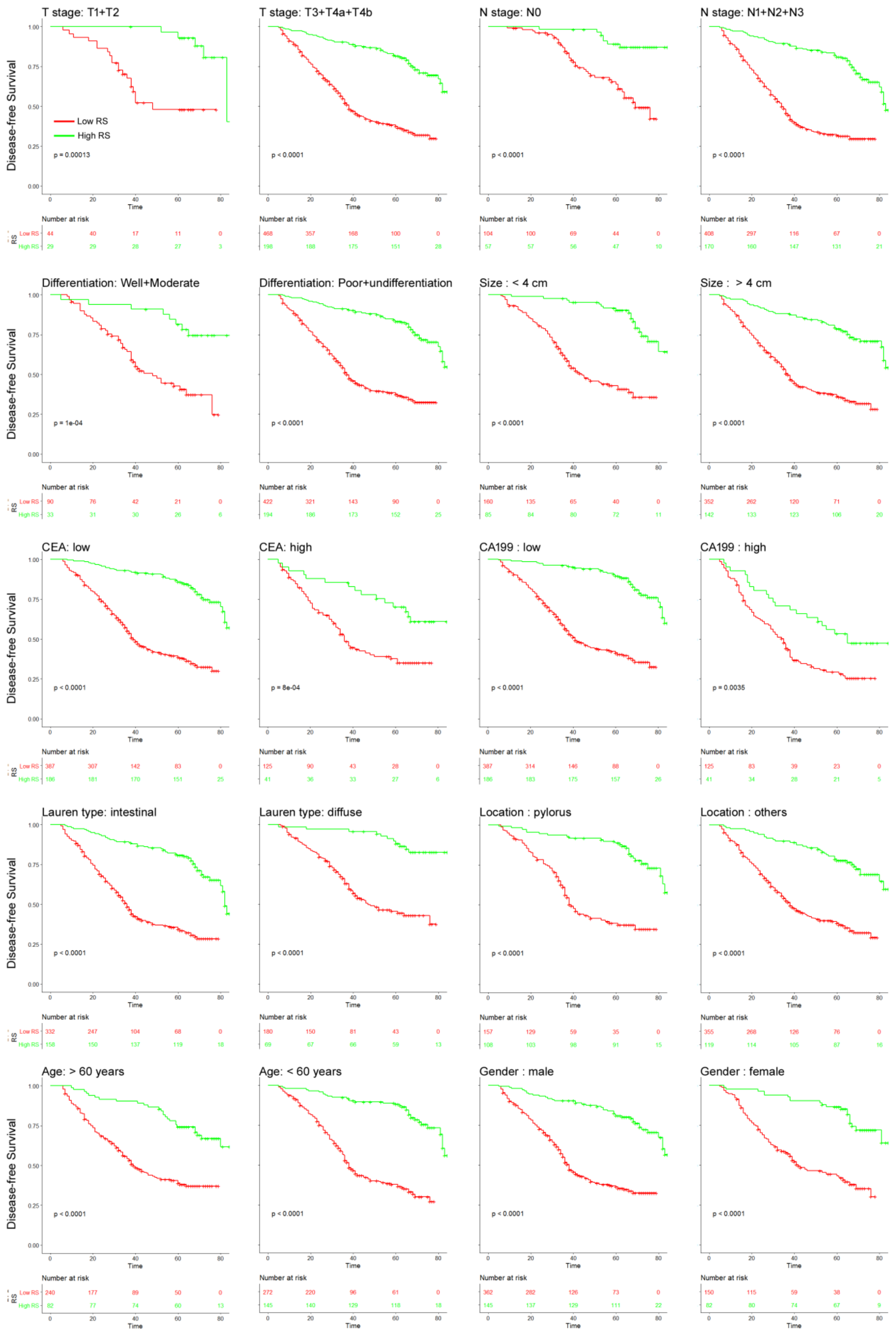

Figure S2. Kaplan-Meier survival analysis of DFS for 739 patients in the validation cohort according to the RS stratified by clinicopathological risk factors. *P*-values were calculated by log-rank test.

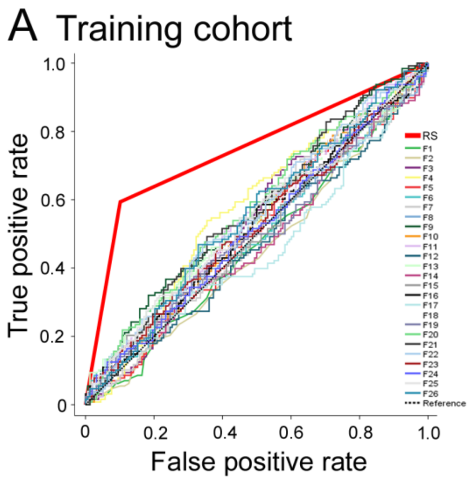

Figure S3. Receiver operating characteristic (ROC) curves of 5-year disease-free survival for the 26 selected features and RS in the training and validation cohorts. RS, radiomic score.

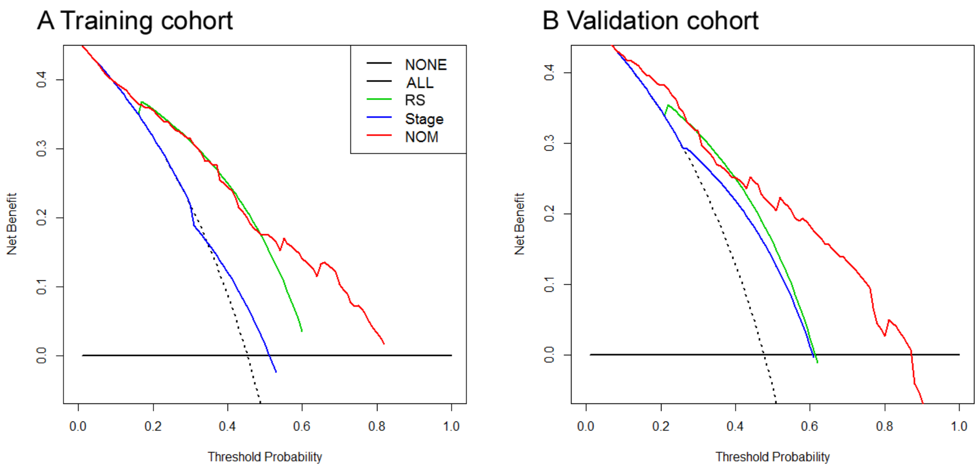

Figure S4. Decision curve analysis of each model for predicting the survival in the training and validation cohorts. (A), training cohort; (B), validation cohort. The y-axis measures the net benefit, and the red line represents integrated nomograms. RS, radiomic score.

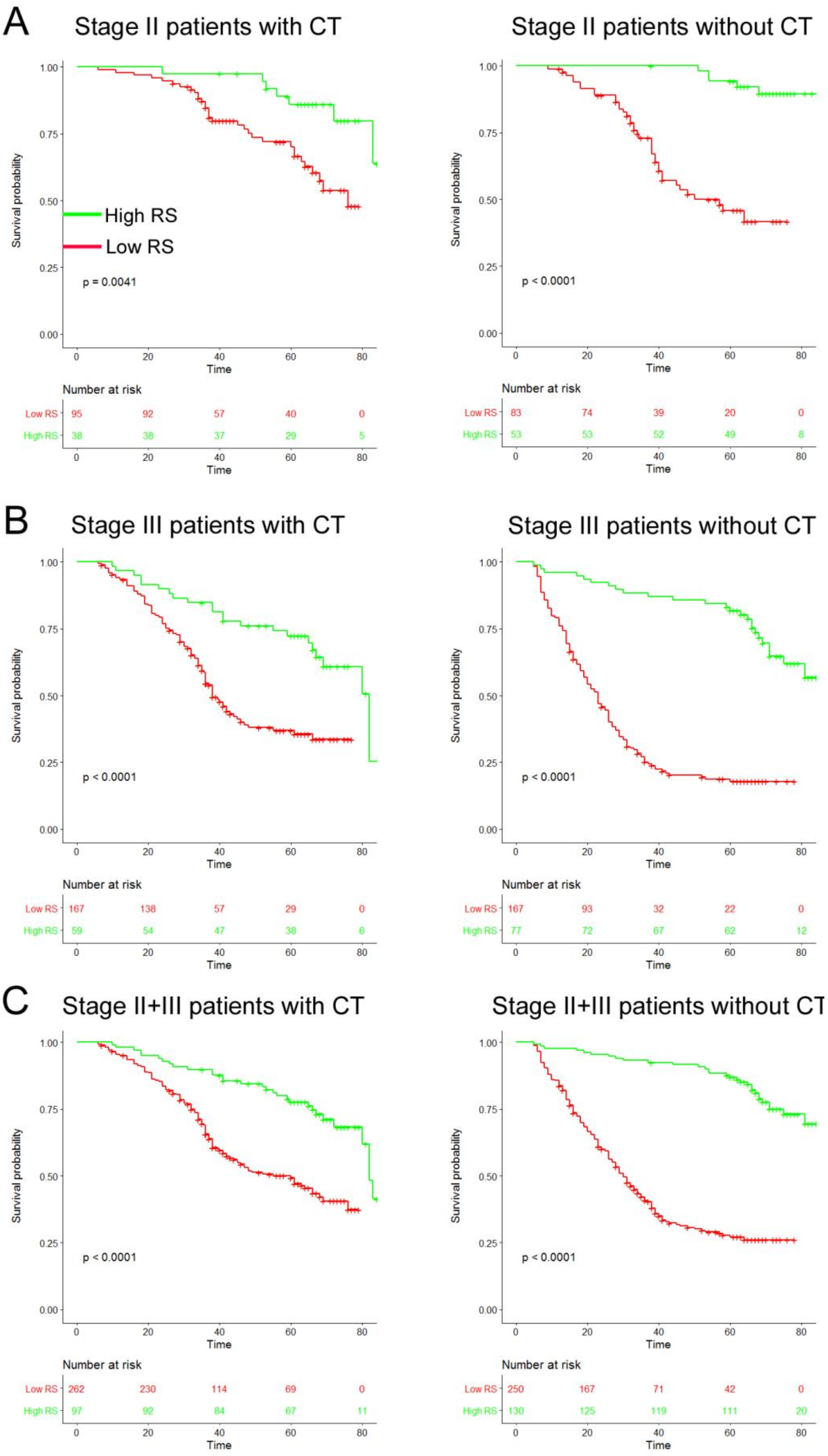

Figure S5. Kaplan-Meier analysis of disease-free survival in patients received postsurgical chemotherapy (CT) according to Rad-score (RS). Left panel: CT patients; right panel: no CT patients. (A) training cohort (n = 286), (B) validation cohort (n = 453), (C) combined cohort (n = 739). *P* -value was calculated by log-rank test.

Table S1. Clinical characteristics of patients according to the SVM signature in the training and validation cohorts.

Variables	Training cohort (n = 223)			Validation cohort (n = 218)		
	low SVM	high SVM	*P*	low SVM	high SVM	*P*
**Gender**			0.353			0.555
Male	103(62.8)	61(37.2)		79(57.7)	58(42.3)	
Female	33(55.9)	26(44.1)		50(61.7)	31(38.3)	
**Age(years), median(IQR)**	57 (49-65)	58 (51-63)		55 (46-63)	54 (49-64)	
**Age(years)**			0.496			0.85
＜60	75(59.1)	52(40.9)		81(58.7)	57(41.3)	
≧60	61(63.5)	35(36.5)		48(60.0)	32(40.0)	
**Tumor size(cm)**			0.129			0.011
＜4	64(56.1)	50(43.9)		39(48.1)	42(51.9)	
≧4	72(66.1)	37(33.9)		90(65.7)	47(34.3)	
**Tumor location**			0.064			0.514
Cardia	26(59.1)	18(40.9)		23(59.0)	16(41.0)	
Body	24(72.7)	9(27.3)		21(51.2)	20(48.8)	
Antrum	63(54.3)	53(45.7)		63(59.4)	43(40.6)	
Whole	23(76.7)	7(23.3)		22(68.8)	10(31.3)	
**Differentiation status**			0.004			0.273
Well+moderate	56(51.4)	53(48.6)		47(54.7)	39(45.3)	
Poor and undifferentiated	80(70.2)	34(29.8)		82(62.1)	50(37.9)	
**Lauren type**			0.057			0.165
Intestinal type	103(57.9)	75(42.1)		94(62.3)	57(37.7)	
Diffuse type	33(73.3)	12(26.7)		35(52.2)	32(47.8)	
**CEA**			0.118			0.68
Elevated	41(69.5)	18(30.5)		26(56.5)	20(43.5)	
Normal	95(57.9)	69(42.1)		103(59.9)	69(40.1)	
**CA199**			0.099			0.296
Elevated	47(69.1)	21(30.9)		34(65.4)	18(34.6)	
Normal	89(57.4)	66(42.6)		95(57.2)	71(42.8)	
**Depth of invasion**			0.042			0.005
T1	5(45.5)	6(54.5)		4(36.4)	7(63.6)	
T2	15(48.4)	16(51.6)		10(41.7)	14(58.3)	
T3	9(64.3)	5(35.7)		9(40.9)	13(59.1)	
T4a	72(58.5)	51(41.5)		65(60.7)	42(39.3)	
T4b	35(79.5)	9(20.5)		41(75.9)	13(24.1)	
**Lymph node metastasis**			0.047			0.022
N0	14(40.0)	21(60.0)		24(47.1)	27(52.9)	
N1	28(57.1)	21(42.9)		23(50.0)	23(50.0)	
N2	56(66.7)	28(33.3)		35(60.3)	23(39.7)	

N3a	29(70.7)	12(29.3)		30(71.4)	12(28.6)	
N3b	9(64.3)	5(35.7)		17(81.0)	4(19.0)	
**Stage**			<0.0001			0.001
II	32(42.7)	43(57.3)		31(43.1)	41(56.9)	
III	104(70.3)	44(29.7)		98(67.1)	48(32.9)	
**Chemotherapy**			0.329			0.509
No	84(63.6)	48(36.4)		58(61.7)	36(38.3)	
Yes	52(57.1)	39(42.9)		71(57.3)	53(42.7)	

Table S2. Univariable analysis of the RS-SVM classifier, patient characteristics and disease-free survival in the training and validation cohorts.

Variable	Training cohort		Validation cohort	
	HR (95%CI)	*P*	HR (95%CI)	*P*
Age(years) (≥60 vs. <60)	1.110 (0.783-1.574)	0.558	1.180 (0.910-1.530)	0.212
Gender (male vs. female)	1.257 (0.849-1.862)	0.252	1.161 (0.877-1.539)	0.297
Tumor size (>4 cm vs. ≤4 cm)	1.727 (0.808-1.173)	0.005	1.201 (0.903-1.596)	0.208
Tumor location	0.973 (0.973-1.364)	0.777	1.160 (1.014-1.327)	0.03
Differentiation status	1.226 (0.783-1.922)	0.373	1.036 (0.723-1.486)	0.846
Lauren type	1.728 (1.185-2.520)	0.004	1.234 (0.921-1.651)	0.158
CEA(ng/ml) (elevated vs. normal)	1.575 (1.083-2.289)	0.017	1.143 (0.834-1.568)	0.406
CA199(U/ml) (elevated vs. normal)	1.962 (1.302-2.956)	0.001	1.754 (1.333-2.308)	<0.0001
Depth of invasion	1.372 (1.179-1.598)	<0.0001	1.269 (1.125-1.430)	<0.0001
Lymph node metastasis	1.424 (1.236-1.639)	<0.0001	1.497 (1.352-1.658)	<0.0001
Stage (III vs. II)	2.069 (1.386-3.088)	<0.0001	3.082 (2.250-4.222)	<0.0001
RS-SVM (high vs. low)	0.190 (0.112-0.324)	<0.0001	0.252 (0.177-0.360)	<0.0001

Table S3. Clinical characteristics of patients according to the chemotherapy in stage II and III patients.

Variables	Number	No chemotherapy (N=380)		Chemotherapy (N=359)		*P*-value
		No.	%	No.	%	
**Gender**						0.716
Female	232	117	50.43%	115	49.57%	
Male	507	263	51.87%	244	48.13%	
**Age(years), median(IQR)**		58(51-65)		57(48-65)		
**Age(years)**						0.51
＜60	417	210	50.36%	207	49.64%	
≧60	322	170	52.80%	152	47.20%	
**Tumor size(cm)**						0.272
＜4	245	133	54.29%	112	45.71%	
≧4	494	247	50.00%	247	50.00%	
**Tumor location**						0.69
Cardia	293	151	51.54%	142	48.46%	
Body	146	69	47.26%	77	52.74%	
Antrum	265	141	53.21%	124	46.79%	
Whole	35	19	54.29%	16	45.71%	
**Differentiation status**						0.882
Well+Moderate	123	64	52.03%	59	47.97%	
Poor and undifferentiated	616	316	51.30%	300	48.70%	
**Lauren type**						0.433
Intestinal type	249	123	49.40%	126	50.60%	
Diffuse or mixed type	490	257	52.45%	233	47.55%	
**CEA**						0.95
Normal	573	295	51.48%	278	48.52%	
Elevated	166	85	51.20%	81	48.80%	
**CA199**						0.32
Normal	573	289	50.44%	284	49.56%	
Elevated	166	91	54.82%	75	45.18%	
**Depth of invasion**						0.001
T1	16	7	43.75%	9	56.25%	
T2	57	29	50.88%	28	49.12%	
T3	200	59	29.50%	141	70.50%	
T4a	413	254	61.50%	159	38.50%	
T4b	53	31	58.49%	22	41.51%	
**Lymph node metastasis**						0.162
N0	161	91	56.52%	70	43.48%	
N1	145	68	46.90%	77	53.10%	
N2	157	73	46.50%	84	53.50%	

N3a	190	97	51.05%	93	48.95%	
N3b	86	51	59.30%	35	40.70%	
**Stage**						0.722
II	269	136	50.56%	133	49.44%	
III	470	244	51.91%	226	48.09%	

Definition of image features

- ***Intensity features-14***

where p(i) is the probability of occurrence of voxels with intensity i

1. CT_min: minimum of CT image
2. CT_max: maximum of CT image
3. CT_mean: mean of CT image
4. CT_median: median of CT image
5. CT_std: Standard Deviation of CT image
6. Hist_Skewness:
   the asymmetry of the grey-level distribution in the histogram
7. Hist_Kurtosis:
   reflects the shape of the grey-level distribution (peaked or flat) relative to a normal distribution.
8. Hist_Entropy:

$$entropy\_hist = -\sum_{i=1} p(i)\log_2[p(i)]$$

9. Hist_Consistency:

$$consistency\_hist = -\sum_{i=1} p(i)^2$$

10. Hist_Energy:

$$energy\_hist = \sum_{i=1} p(i)^2$$

11. CT_range: the range of CT image
12. Hist_Var:
    Variance of CT image
13. CT_RMS: root mean square, the quadratic mean, or the square root of the mean of squares of CT image

14. CT_MAD: Mean absolute deviation, the mean of the absolute deviations of CT image around the mean CT value.

- ***Shape features-8***

Shape features, describing the shape and size of the volume of interest. Let M as the number

of voxels in the tumor.

Using “regionprops” in Matlab.

15. Area: Actual number of pixels in the region, returned as a scalar.
16. Orientation: Angle between the x-axis and the major axis of the ellipse that has the same second-moments as the region, returned as a scalar. The value is in degrees, ranging from -90 degrees to 90 degrees.
17. Eccentricity: Eccentricity of the ellipse that has the same second-moments as the region, returned as a scalar.
18. Equivdiameter: Diameter of a circle with the same area as the region, returned as a scalar.sqrt(4*Area/pi)
19. Solidity: Proportion of the pixels in the convex hull that are also in the region, returned as a scalar.
    Area/ConvexArea
20. Extent: Ratio of pixels in the region to pixels in the total bounding box, returned as a scalar.
    Area divided by the area of the bounding box
21. Eulernumber: Number of objects in the region minus the number of holes in those objects.
22. Perimeter: Distance around the boundary of the region.

- ***Gray Level Co-occurrence Matrix-based features (GLCM)-23***

Gray level co-occurrence matrix-based features, as described by study. The element $P(i,j)$ of normalized co-occurrence matrix represent the number of times that intensity i and j appeared in two voxels separated by distance D in direction θ. The co-occurrence matrix is given by:

$$P(i,j) = \#\{(I(x,y,z) = i, I(k,l,m) = j) \mid D, \theta\}$$

where # represents the number of times, I represents the voxel intensity, (x,y,z) and (k,l,m) are the coordinates (positions) of two different voxels, the direction vector is thus determined by $(k,l,m)-(x,y,z)$, N_g is the number of discrete intensity levels in the image, and μ is the mean of $P(i,j)$. The feature is derived by considering all the 13 directions simultaneously, thus arriving at a single matrix.

Let us define:

$$\mu_x = \sum_{i=1}^{N_g} i \sum_{j=1}^{Ng} P(i,j) \qquad \mu_y = \sum_{j=1}^{N_g} j \sum_{i=1}^{Ng} P(i,j)$$

$$\sigma_x = \sqrt{\sum_{i=1}^{N_g} (i-\mu_x)^2 \sum_{j=1}^{Ng} P(i,j)} \qquad \sigma_y = \sqrt{\sum_{j=1}^{N_g} (j-\mu_y)^2 \sum_{i=1}^{Ng} P(i,j)}$$

$$p_x(i)=\sum_{j=1}^{N_g} P(i,j) \qquad p_y(j)=\sum_{i=1}^{N_g} P(i,j).$$

$$p_{x+y}(k)=\sum_{i=1}^{N_g}\sum_{j=1}^{N_g} P(i,j),\ i+j=k, k=2,3,\ldots 2N_g$$

$$p_{x-y}(k)=\sum_{i=1}^{N_g}\sum_{j=1}^{N_g} P(i,j),\ |i-j|=k, k=0,1,\ldots N_g-1$$

$$\mu_{x\text{-}y}=\sum_{k=0}^{N_g-1} kP_{x\text{-}y}(k)$$

$$HXY1=-\sum_{i=1}^{N_g}\sum_{j=1}^{N_g} P(i,j)\log_2\left(p_x(i)\,p_y(j)\right)$$

$$HXY2=-\sum_{i=1}^{N_g}\sum_{j=1}^{N_g} P_x(i)P_y(j)\log_2\left(p_x(i)\,p_y(j)\right)$$

$$HX=-\sum_{i=1}^{N_g} p_x(i)\log_2[p_x(i)] \qquad HY=-\sum_{j=1}^{N_g} p_y(j)\log_2[p_y(j)]$$

$$H=-\sum_{i=1}^{N_g}\sum_{j=1}^{N_g} p(i,j)\log_2[p(i,j)]$$

The various radiomics features based on the co-occurrence matrix are then defined as:

1. Auto correlation (AutoCorrelation):

$$auto\ correlation=\sum_{i=1}^{N_g}\sum_{j=1}^{N_g} ij\,p(i,j)$$

2. Cluster prominence (ClusterPro):

$$cluster\ prominence=\sum_{i=1}^{N_g}\sum_{j=1}^{N_g}\left[i+j-\mu_x-\mu_y\right]^4 P(i,j)$$

3. Cluster shade (ClusterShade):

$$cluster\ shade=\sum_{i=1}^{N_g}\sum_{j=1}^{N_g}\left[i+j-\mu_x-\mu_y\right]^3 P(i,j)$$

4. Cluster tendency (ClusterTen):

$$cluster\ tendency=\sum_{i=1}^{N_g}\sum_{j=1}^{N_g}\left[i+j-\mu_x-\mu_y\right]^2 P(i,j)$$

5. Contrast:

$$contrast=\sum_{i=1}^{N_g}\sum_{j=1}^{N_g}|i-j|^2 P(i,j)$$

6. Correlation:

$$correlation = \sum_{i=1}^{N_g}\sum_{j=1}^{N_g}\frac{(i-\mu_x)(j-\mu_y)p(i,j)}{\sigma_x\sigma_y} = \frac{\sum_{i=1}^{N_g}\sum_{j=1}^{N_g} ijP(i,j)-\mu_x\mu_y}{\sigma_x\sigma_y}$$

7. Difference entropy (DiffEntropy):

$$difference\ entropy = -\sum_{k=0}^{N_g-1} P_{x-y}(k)\log_2\left[P_{x-y}(k)\right]$$

8. Difference Variance (DiffVar):

$$difference\ variance = -\sum_{k=0}^{N_g-1}\left(k-\mu_{x-y}\right)^2 P_{x-y}(k) \qquad \mu_{x\text{-}y} = \sum_{k=0}^{N_g-1} kP_{x\text{-}y}(k)$$

9. Dissimilarity:

$$dissimilarity = \sum_{i=1}^{N_g}\sum_{j=1}^{N_g}|i-j|P(i,j)$$

10. Energy, called Uniformity in[1], also called Angular second moment in[1]:

$$energy = \sum_{i=1}^{N_g}\sum_{j=1}^{N_g}\left[P(i,j)\right]^2$$

11. Entropy:

$$entropy = -\sum_{i=1}^{N_g}\sum_{j=1}^{N_g} P(i,j)\log_2\left[P(i,j)\right]$$

12. Homogeneity, also called Inverse difference in[1] :

$$homogeneity = \sum_{i=1}^{N_g}\sum_{j=1}^{N_g}\frac{P(i,j)}{1+|i-j|}$$

13. Informational measure of correlation 1 (IMC1):

$$IMC1 = \frac{H-HXY1}{\max\{HX,HY\}}$$

Where *HX and HY* are the entropies of $p_x(i)$ and $p_y(j)$.

14. Informational measure of correlation 2 (IMC2):

$$IMC2 = \sqrt{1-e^{-2(HXY2-H)}}$$

where *H* is the entropy of $p(i,j)$.

15. Inverse Difference Normalized (IDN):

$$IDN = \sum_{i=1}^{N_g} \sum_{j=1}^{N_g} \frac{P(i,j)}{1 + \left(\frac{|i-j|}{N} \right)}$$

16. Inverse Difference Moment Normalized (IDMN):

$$IDMN = \sum_{i=1}^{N_g} \sum_{j=1}^{N_g} \frac{P(i,j)}{1 + \left(\frac{|i-j|^2}{N^2} \right)}$$

17. Inverse variance (InVar):

$$inverse\ variance = \sum_{i=1}^{N_g} \sum_{j=1}^{N_g} \frac{P(i,j)}{|i-j|^2}, i \neq j$$

18. Maximum probability (MaxPossilility):

$$maximum\ probability = \max\{P(i,j)\}$$

19. Sum average2:

$$sum\ average2 = \sum_{k=2}^{2N_g} \left[kP_{x+y}(k) \right]$$

20. Sum entropy (SumEntropy):

$$sum\ entropy = -\sum_{k=2}^{2N_g} P_{x+y}(k) \log_2 \left[P_{x+y}(k) \right]$$

21. Sum variance (SumVar):

$$sum\ variance = \sum_{k=2}^{2N_g} (k - SA)^2 P_{x+y}(k)$$

where SA is Sum average2.

22. Variance:

$$variance = \frac{1}{N_g \times N_g} \sum_{i=1}^{N_g} \sum_{j=1}^{N_g} [(i-\mu_x)^2 + (j-\mu_y)^2] p(i,j)$$

- ***Gray Level Run Length Matrix-based features (GLRLM)-13***

Gray level run length matrix-based features are described by Galloway et al.[3]. The element of GLRLM $P(i,j)$ counts the number of runs j with collinearly adjacent pixels having the same

gray level intensity i as follows:

$$P(i,j)=\left\{j \mid I_1=i, I_2=i, \ldots, I_j=i\right\}$$

where $I_1, I_2, \ldots, I_j$ are collinearly adjacent voxels.

The GLRLM feature value was derived by considering all the 13 directions simultaneously, thus arriving at a single matrix. Let $P(i,j)$ be the $(i,j)\,th$ entry in the given run-length matrix, N_g the number of discrete intensity values in the image, N_r the number of different run lengths, N_p the number of voxels in the image, and the entry (i,j) of the normalized GLRLM defined as:

$$p(i,j)=\frac{P(i,j)}{\sum_{i=1}^{N_g}\sum_{j=1}^{N_r}P(i,j)} \quad \mu_i=\sum_{i=1}^{N_g} i\sum_{j=1}^{N_r} p(i,j) \quad \mu_j=\sum_{j=1}^{N_r} j\sum_{i=1}^{N_g} p(i,j)$$

Then the GLRLM-based features are defined as:

1. Short Run Emphasis (SRE):

$$SRE=\sum_{i=1}^{N_g}\sum_{j=1}^{N_r}\left[\frac{p(i,j)}{j^2}\right]$$

2. Long Run Emphasis (LRE):

$$LRE=\sum_{i=1}^{N_g}\sum_{j=1}^{N_r} j^2 p(i,j)$$

3. Gray Level Non-Uniformity (GLN):

$$GLN=\sum_{i=1}^{N_g}\left[\sum_{j=1}^{N_r} p(i,j)\right]^2$$

4. Run Length Non-Uniformity (RLN):

$$RLN=\sum_{j=1}^{N_r}\left[\sum_{i=1}^{N_g} p(i,j)\right]^2$$

5. Run Percentage (RP):

$$RP=\sum_{i=1}^{N_g}\sum_{j=1}^{N_r}\frac{p(i,j)}{N_p}$$

6. Low Gray Level Run Emphasis (LGRE):

$$LGRE=\sum_{i=1}^{N_g}\sum_{j=1}^{N_r}\left[\frac{p(i,j)}{i^2}\right]$$

7. High Gray Level Run Emphasis (HGRE):

$$HGRE=\sum_{i=1}^{N_g}\sum_{j=1}^{N_r} i^2 p(i,j)$$

8. Short Run Low Gray Level Emphasis (SRLGE):

$$SRLGE = \sum_{i=1}^{N_g}\sum_{j=1}^{N_r}\left[\frac{p(i,j)}{i^2 j^2}\right]$$

9. Short Run High Gray Level Emphasis (SRHGE):

$$SRHGE = \sum_{i=1}^{N_g}\sum_{j=1}^{N_r}\left[\frac{p(i,j)i^2}{j^2}\right]$$

10. Long Run Low Gray Level Emphasis (LRLGE):

$$LRLGE = \sum_{i=1}^{N_g}\sum_{j=1}^{N_r}\left[\frac{p(i,j)j^2}{i^2}\right]$$

11. Long Run High Gray Level Emphasis (LRHGE):

$$LRHGE = \sum_{i=1}^{N_g}\sum_{j=1}^{N_r} p(i,j)i^2 j^2$$

12. Gray Level Variance (GLV)

$$GLV = \frac{1}{N_g \times N_r}\sum_{i=1}^{N_g}\sum_{j=1}^{N_r}(ip(i,j)-\mu_i)^2$$

13. Run length Variance (RLV)

$$RLV = \frac{1}{N_g \times N_r}\sum_{i=1}^{N_g}\sum_{j=1}^{N_r}(jp(i,j)-\mu_j)^2$$

- ***Gray Level Size Zone Matrix-based features (GLSZM)-13***

Gray-level size-zone matrix-based features, was described in [1]. GLSZM describes the number of a certain size zone j having same intensity i within N-connected neighbors in a 3D space as follows:

$$P(i,j) = \left\{ j \mid I_1 = i, I_2 = i, \ldots, I_j = i \right\}$$

where voxels $I_1, I_2, \ldots, I_j$ are within N-connected neighbors (N=26).

Let $P(i,j)$ be the $(i,j)th$ entry in the given size-zone matrix, N_g the number of discrete intensity values in the image, N_z the size of the largest homogeneous region in the volume of interest, and N_α the number homogeneous zones in the image. The entry (i,j) of the GLSZM are then normalized as:

$$p(i,j) = \frac{P(i,j)}{\sum_{i=1}^{N_g}\sum_{j=1}^{N_z} P(i,j)} \qquad \mu_i = \sum_{i=1}^{N_g} i \sum_{j=1}^{N_z} p(i,j) \qquad \mu_j = \sum_{j=1}^{N_z} j \sum_{i=1}^{N_g} p(i,j)$$

The GLSZM-based features are then defined as:

1. Small Zone Emphasis (SZE):

$$SZE = \sum_{i=1}^{N_g} \sum_{j=1}^{N_z} \left[\frac{p(i,j)}{j^2} \right]$$

2. Large Zone Emphasis (LZE):

$$LZE = \sum_{i=1}^{N_g} \sum_{j=1}^{N_z} j^2 p(i,j)$$

3. Gray Level Non-uniformity (GLN) also called Intensity Variability (IV) in[4]:

$$GLN = \sum_{i=1}^{N_g} \left[\sum_{j=1}^{N_z} p(i,j) \right]^2$$

4. Zone Size Non-uniformity (ZSN) also called Size Zone Variability (SZV) in[4]:

$$ZSN = \sum_{j=1}^{N_z} \left[\sum_{i=1}^{N_g} p(i,j) \right]^2$$

5. Zone Percentage (ZP):

$$ZP = \sum_{i=1}^{N_g} \sum_{j=1}^{N_z} \frac{p(i,j)}{N_\alpha}$$

6. Low Gray Level Zone Emphasis (LGZE) also called Low Intensity Emphasis (LIE) in[4]:

$$LGZE = \sum_{i=1}^{N_g} \sum_{j=1}^{N_z} \left[\frac{p(i,j)}{i^2} \right]$$

7. High Gray level Zone Emphasis (HGZE) also called High Intensity Emphasis (HIE) in[4]:

$$HGZE = \sum_{i=1}^{N_g} \sum_{j=1}^{N_z} i^2 p(i,j)$$

8. Small Zone Low Gray Level Emphasis (SZLGE) also called Low Intensity Small Area Emphasis (LISAE) in[4]:

$$SZLGE = \sum_{i=1}^{N_g} \sum_{j=1}^{N_z} \left[\frac{p(i,j)}{i^2 j^2} \right]$$

9. Small Zone High Gray-Level Emphasis (SZHGE) also called High Intensity Small Area Emphasis (HISAE) in[4]:

$$SZHGE = \sum_{i=1}^{N_g} \sum_{j=1}^{N_z} \left[\frac{p(i,j) i^2}{j^2} \right]$$

10. Large Zone Low Gray-Level Emphasis (LZLGE) also called Low Intensity Large Area Emphasis (LILAE) in[4]:

$$LZLGE = \sum_{i=1}^{N_g} \sum_{j=1}^{N_z} \left[\frac{p(i,j) j^2}{i^2} \right]$$

11. Large Zone High Gray-Level Emphasis (LZHGE) also called High Intensity Large Area Emphasis (HILAE) in[4]:

$$LZHGE = \sum_{i=1}^{N_g} \sum_{j=1}^{N_z} p(i,j) i^2 j^2$$

12. Gray Level Variance (GLV)

$$GLV = \frac{1}{N_g \times N_z} \sum_{i=1}^{N_g} \sum_{j=1}^{N_z} (ip(i,j) - \mu_i)^2$$

13. Zone Size Variance (ZSV)

$$ZSV = \frac{1}{N_g \times N_z} \sum_{i=1}^{N_g} \sum_{j=1}^{N_z} (jp(i,j) - \mu_j)^2$$

where zone aforesaid also called area in[4].

- ***Neighborhood Gray Tone Difference Matrix–based features (NGTDM)-5***

NGTDM is a column matrix[5]. Denote the ith entry of the NGTDM as $P(i)$, defined as:

$$P(i) = \begin{cases} \sum_{i \in \{N_i\}} \left| i - \overline{A_i} \right| & if\ N_i > 0, \\ 0 & otherwise. \end{cases}$$

where $\{N_i\}$ is the set of all voxels with gray-level i in tumor volume (including the peripheral region), N_i is the number of voxels with gray-level i in tumor volume, and A_i is the average gray level of the M connected neighbors around a center voxel $V(i,j,k)$ with gray level i. Also, we have

$$\overline{A_i} = \overline{A}(j,k,l) = \frac{1}{M} \sum_{m=-d}^{d} \sum_{n=-d}^{d} \sum_{s=-d}^{d} V(j+m, k+n, l+s), (m,n,l) \neq (0,0,0)$$

where $d = 1$, specifies the window size as $3 \times 3 \times 3$, and $M = (2d+1)^3 - 1$. The quantity $n_i = \frac{N_i}{N}$ is also defined, where N is the total number of voxels in tumor volume. The NGTDM-based features are then defined as:

1. Coarseness:

$$coarseness = [\varepsilon + \sum_{i=1}^{N_g} n_i P(i)]^{-1}$$

where ε is a small number to prevent coarseness becoming infinite, N_g the number of discrete

intensity values in the image.

2. Contrast:

$$contrast = [\frac{1}{N_g \times (N_g - 1)} \sum_{i=1}^{N_g} \sum_{j=1}^{N_g} n_i n_j (i-j)^2] \times [\frac{1}{N} \sum_{i=1}^{N_g} P(i)]$$

3. Busyness:

$$busyness = \frac{\sum_{i=1}^{N_g} n_i P(i)}{\sum_{i=1}^{N_g} \sum_{j=1}^{N_g} \left| i n_i - j n_j \right|}, n_i \neq 0, n_j \neq 0$$

4. Complexity:

$$complexity = \sum_{i=1}^{N_g} \sum_{j=1}^{N_g} \frac{|i-j| \left[n_i P(i) + n_j P(j) \right]}{N(n_i + n_j)}, n_i \neq 0, n_j \neq 0$$

5. Strength:

$$strength = \frac{\sum_{i=1}^{N_g} \sum_{j=1}^{N_g} (n_i + n_j)(i-j)^2}{\varepsilon + \sum_{i=1}^{N_g} P(i)}, n_i \neq 0, n_j \neq 0$$

where ε is a small number to prevent strength becoming infinite.
